# Pan-cancer onco-signatures reveal a novel mitochondrial subtype of luminal breast cancer with specific regulators

**DOI:** 10.1186/s12967-023-03907-z

**Published:** 2023-01-30

**Authors:** Ines Simeone, Michele Ceccarelli

**Affiliations:** 1 Department of Electrical Engineering and Information Technology, University of Naples “Federico II”, Via Claudio 21, 80128 Naples, Italy; 2 Center for Genomic Science of IIT@SEMM, Istituto Italiano di Tecnologia (IIT), Via Adamello 16, 20139 Milan, Italy; 3 BIOGEM Institute of Molecular Biology and Genetics, Via Camporeale, 83031 Ariano Irpino, Italy

**Keywords:** Onco-signatures, Pan-cancer, Breast cancer disease, TCGA, Gene set enrichment analysis, Normalized enrichment score, Luminal breast tumor subtype, EMT, Hsa-miR-135-5p, TDMD

## Abstract

**Background:**

Somatic alterations in cancer cause dysregulation of signaling pathways that control cell-cycle progression, apoptosis, and cell growth. The effect of individual alterations in these pathways differs between individual tumors and tumor types. Recognizing driver events is a complex task requiring integrating multiple molecular data, including genomics, epigenomics, and functional genomics. A common hypothesis is that these driver events share similar effects on the hallmarks of cancer. The availability of large-scale multi-omics studies allows for inferring these common effects from data. Once these effects are known, one can then deconvolve in every individual patient whether a given genomics alteration is a driver event.

**Methods:**

Here, we develop a novel data-driven approach to identify shared oncogenic expression signatures among tumors. We aim to identify gene onco-signature for classifying tumor patients in homogeneous subclasses with distinct prognoses and specific genomic alterations. We derive expression pan-cancer onco-signatures from TCGA gene expression data using a discovery set of 9107 primary pan-tumor samples together with respective matched mutational data and a list of known cancer-related genes from COSMIC database.

**Results:**

We use the derived ono-signatures to state their prognostic significance and apply them to the TCGA breast cancer dataset as proof of principle of our approach. We uncover a “mitochondrial” sub-group of Luminal patients characterized by its biological features and regulated by specific genetic modulators. Collectively, our results demonstrate the effectiveness of onco-signatures-based methodologies, and they also contribute to a comprehensive understanding of the metabolic heterogeneity of Luminal tumors.

**Conclusions:**

These findings provide novel genomics evidence for developing personalized breast cancer patient treatments. The onco-signature approach, demonstrated here on breast cancer, is general and can be applied to other cancer types.

**Supplementary Information:**

The online version contains supplementary material available at 10.1186/s12967-023-03907-z.

## Background

The integration of next-generation sequencing together with other high-throughput techniques has provided an excellent opportunity for the study of molecular alterations occurring in cancer [1, 2]. In particular, the platforms for gene expression profiling have been widely used to identify cancer biomarkers. Over the last few decades, it has been recognized the general idea that a singular alteration can not cause cancer, rather than it was recognized as the result of a wider sequence of genetic and genomic events occurring during the progression from normal epithelial tissue to metastatic disease [3, 4]. For this reason, methodologies based on the use of gene signatures, i.e., lists of genes sharing a common pattern of expression among multiple tumor types, is currently recognized as a more biologically significant approach to understanding the biology of cancer [5].

In the present study, we identified 105 onco-signatures associated with the more frequent mutational events shared among the various cancer types. As proof of principle, we evaluate the power of the derived onco-signatures to classify TCGA breast cancer patients in relevant groups with distinct biology and clinical outcome. In addition, we have successfully identified two different metabolic subtypes of Luminal tumors based on 28 specific breast cancer prognostic onco-signatures.

Here, we propose a novel methodological framework to identify commonly shared onco-signatures in cancer that can contribute to the understanding of the role of alterations in tumor disease and the identification of novel molecular mechanisms useful for developing precision therapeutic strategies.

## Methods

### Data collection

RNA-seq gene expression data, somatic mutation information, and clinical patient annotation of 32 TCGA solid primary tumor type datasets (ACC, BLCA, BRCA, CESC, CHOL, COAD, DLBC, ESCA, GBM, HNSC, KICH, KIRC, KIRP, LGG, LIHC, LUAD, LUSC, MESO, OV, PAAD, PCPG, PRAD, READ, SARC, SKCM, STAD, TCGT, THCA, THYM, UCEC, UCS, UVM) were retrieved from the Genomic Data Commons (GDC) Data Portal (https://portal.gdc.cancer.gov) using the R/Bioconductor package TCGAbiolinks [6]. Gene count RNA-seq data, including 9107 primary tumor samples, was normalized by using both within-lane gene-level GC-content and full/upper-quantile normalization methods [7]. The Catalogue of Somatic Mutations in Cancer (COSMIC) database (https://cancer.sanger.ac.uk/cosmic/curation) was used to obtain the list of genes containing mutations implicated in cancer disease (N = 277). The public BRCA loci-based isoform.quantification.txt file, which reports raw and normalized counts of every distinct small RNA-seq observed, was downloaded from TCGA by using TCGAbiolinks [6]. The expression level of each mature miRNA was then calculated as the sum of all isoforms corresponding to the same unique miRBase (https://www.mirbase.org) MIMAT identifier (MIMAT-id). Next, each MIMAT-id was translated into a miRBase name using the miRNA Converter tool of MiRandola database [8]. TCGA BRCA GISTIC2.0 [9] all_thresholded_by_genes.txt output data file, which reports a score to indicate if a gene is considered undergoing homozygous deletion, copy number loss, copy number gain and/or amplification (respectively scores equal to − 2, − 1, 0, 1, 2), was downloaded from the GDAC firehose web portal (https://gdac.broadinstitute.org) by using the Bioconductor RTCGA Toolbox package [10]. Whereas TCGA DNA methylation beta values always related to breast cancer disease were retrieved by using the R/Bioconductor package TCGAbiolinks [6].

### Onco-signatures derivation

For each cancer-related gene annotated in COSMIC data annotation, differentially expressed genes (DEGs) between the mutated (MUT) and wild-type (WT) samples were identified using the bi.deg function of DEComplexDisease R package [10]. DeComplexDisease is a tool built to find the differential expressed genes for phenotypes characterized by heterogeneous genomic expression profiles like complex diseases. DeComplexDisease applies a bi-clustering algorithm to find the genes shared by patients associated with complex phenotypes, which is why they are affected by the identical altered molecular mechanism. As the first step DEComplexDisease applies the bi.deg function, which transforms the RNA-seq counts or normalized expression matrix into binary differential expression matrix of − 1, 0, and 1, which indicates respectively the down-regulation, no change, and up-regulation. There are three main steps: (i) the normal samples are used to construct the expression references and estimate two parameters of the distribution, which are mean and dispersion for RNA-seq counts (modeled by a negative-binomial distribution) or mean and standard deviation for normalized or microarray gene expression data; (ii) for every gene *i* in every sample *j* of the disease matrix *x*_ij_, it computes the probability that a random value of the estimated distribution gets a value equal or greater than *x*_ij_; (iii) using the p-value cutoff defined by the users (0.05 in our experiments), the bi.deg function assigns 1 or − 1 to indicate the up- or down-regulated genes, where 1 is the up-regulated genes and − 1 is the down-regulated genes. The other genes are assigned with 0. This is the final output DEGs matrix of the For each derived binary DEGs matrix, the total number of mutated samples showing − 1 or 1 was determined, gene by gene, to estimate the quantile distribution of counts and select the DEGs most commonly shared across the mutated samples, and so more associated to the mutated phenotype, that for us are the DEGs falling above the 98th percentile of the quintile distribution of counts. Next, unique marker genes were identified for each derived onco-signature and only the signatures with at least five marker genes were selected for further analyses (N = 105).

### Normalized enrichment score (NES) estimation and NES clustering analysis of breast cancer survival-associated onco-signatures

Cox’s proportional hazards regression analysis was performed to assess the prognostic ability of our collection of 105 onco-signatures in primary TCGA breast cancer samples to identify the most significant (p-value < 0.05) onco-signatures associated with survival in breast cancer disease. Next, gene-sets enrichment analysis was performed to estimate the normalized enrichment score (NES) of the more relevant breast cancer survival-associated onco-signatures. In particular, the analysis was carried out by interrogating the gene expression values profiled by RNA sequencing of 1093 primary breast cancer tumor samples from TCGA using the mwwGST function of yaGST R package, which runs a competitive single-sample Mann–Whitney–Wilcoxon gene set test [11]. NES is an estimate of the probability that the expression of a gene in the geneset is greater than the expression of a gene outside this set:

${NES}= 1-\frac{U}{mn}$ where *m* is the number of genes in a gene set, *n* is the number of those outside the gene set, ${U}={mn}+{m(m+1)} -{T}$ and *T* is the sum of the ranks of the genes in the geneset. Then, the matrix of NES concerning the 1093 TCGA breast cancer samples was used to calculate distances between onco-signatures’ gene-sets to build a hierarchical clustering, using as clustering parameters ward criterion (ward.D2 method) and the number of clusters (k) equal to 4.

### Differential expression analysis and gene ontology enrichment analysis

The TCGAanalyze_DEA function of TCGAbiolinks R/Bioconductor package [6] was used to perform differential expression analysis by applying the edgeR method [12]. The clusterProfiler R package [13] was used for Gene Ontology (GO) enrichment analysis by its gseGO function. The dotplot function of enrichplot R package [14] was used to visualize functional enrichment results. Normalization of the gene expression matrix was performed using the EDASeq approach [7]. The miRNA Enrichment Analysis and Annotation Tool (miEAA, https://ccb-compute2.cs.uni-saarland.de/mieaa2/) was used to perform over-representation analysis (ORA) of differentially expressed microRNAs (DEmiRs).

### Transcription factors motif discovery

Promoter sequences (1500 nucleotides upstream of gene transcription start site (TSS) and 500 nucleotides downstream of TSS) of MIR135A1 and MIR135A2 genes were retrieved from UCSC (https://genome.ucsc.edu) by using R/Bioconductor packages BSgenome.Hsapiens.UCSC.hg19 [15] and Biostrings [16]. MotifDb package available in Bioconductor [17] was used to search and retrieve in JASPAR database (https://jaspar.genereg.net/) DNA-binding motifs of known transcription factors. Then, for each JASPAR transcription factor motif, the possible matches in the promoters of the two genes coding for hsa-miR-135a-5p were identified by using the matchPWM function of the Biostrings package [16].

### Tumor microenvironment infiltration estimation and chemotherapeutic sensitivity prediction

The abundance of six tumor-infiltrating immune cells subsets (B cells, CD4 T cells, CD8 T cells, macrophages, neutrophils, and dendritic cells) was estimated by using TIMER2.0 webserver (http://timer.cistrome.org) [18]. TIMER2.0 provides immune infiltrates’ abundances estimation by multiple immune deconvolution algorithms (TIMER [19, 20], CIBERSORT [21], quanTIseq [22], xCell [23], MCP-counter [24], and EPIC [25]). In addition, pRRophetic R package [26] was used to predict the clinical chemotherapeutic response of the two different metabolic Luminal breast cancer groups to 138 known anticancer drugs. Wilcoxon rank sum test was then used to compare the half-maximal inhibitory concentration (IC50) differences between the two groups in comparison.

## Results

### Onco-signatures identify four distinct phenotypes of breast cancer

**Fig. 1:**
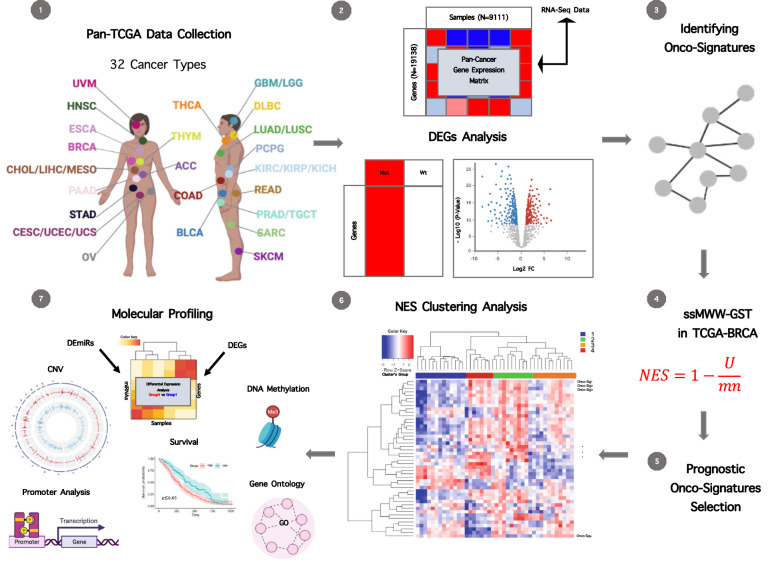
The whole workflow chart of the study. Seven processes include as follows: ① Pan-cancer data collection from TCGA, ② Performing differential gene expression analysis (mutated versus wild type samples) for each cancer related genes annotated in COSMIC database, ③ Identification of 105 onco-signatures, each one composed by specific marker genes, ④ Performing of the single-sample Mann–Whitney–Wilcoxon gene set test in TCGA-BRCA dataset, ⑤ Identification of the 28 most prognostic breast cancer onco-signatures, by applying the Cox proportional hazard model, ⑥ Performing the clustering analysis on the matrix of normalized enrichment scores calculated through the single-sample Mann–Whitney–Wilcoxon gene set test, showing on the rows the 28 breast survival associated onco-signatures and on the columns the TCGA primary breast cancer samples, ⑦ Molecular characterization of the identified groups through survival analysis, differential expression analysis, copy number variation analysis, DNA methylation analysis, promoter analysis, gene ontology enrichment analysis

Cancer is caused by an accumulation of somatic mutations in genes involved in important biological processes like cellular growth or DNA repair activity [3, 4]. Due to the specificity of the mutational event, its penetrance, the compensation mechanisms deployed by the cell to reduce the impact of the injury, and to the cancer environment, the molecular lesion can generate new patterns of gene expression that can be shared among cancer types and driver events [27]. To identify common cancer-relevant molecular tissue-agnostic patterns induced by recurrent mutations, we developed a novel integrative procedure summarized in Fig. 1. A total of 105 onco-signatures were derived by using a pan-cancer approach (Additional file 1: Table S1). We evaluated the impact of derived onco-signatures only in breast cancer type. We applied the Cox proportional hazard model using the survival data of 1093 primary breast cancer patients from TCGA to identify the onco-signatures whose activities are significantly (p < 0.05) associated with the survival, selecting in this way a total of 28 breast cancer survival associated onco-signatures. Next, we tested the ability of the selected 28 breast cancer prognostic onco-signatures to stratify the 1093 TCGA primary breast cancer samples by clustering the normalized enrichment score values obtained for each onco-signature in each tumor sample.

**Fig. 2:**
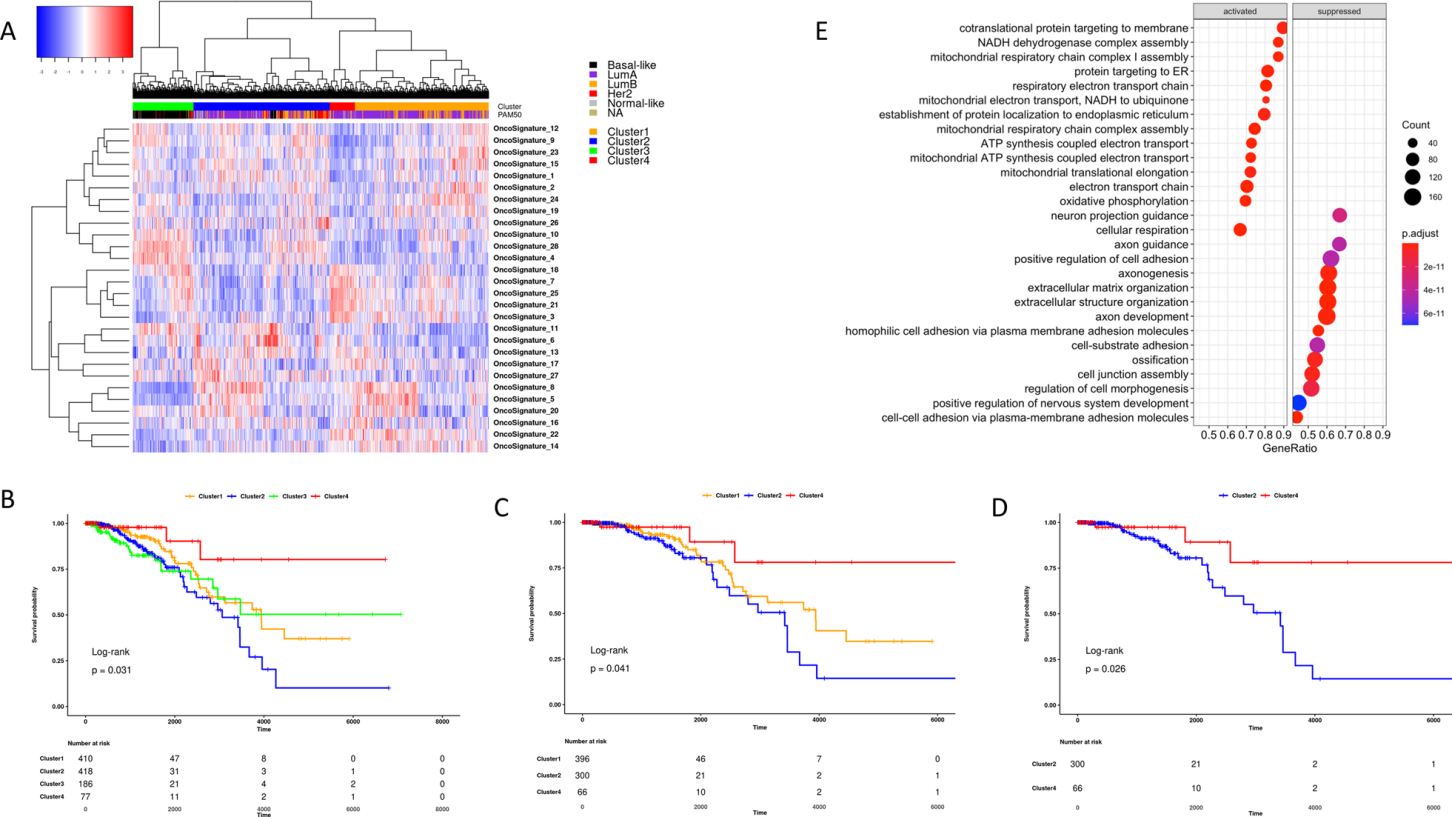
NES clustering analysis defines four phenotypes of primary breast cancer with different survival outcomes. **A** Unsupervised hierarchical cluster (Ward.D2 criterion) of primary TCGA breast cancer samples applied to the matrix of the enrichment scores calculated through the single-sample Mann–Whitney–Wilcoxon gene set test for each identified mammary tumor prognostic onco-signature. In red the onco-signatures significantly enriched, and in blue those significantly depleted across the four clusters. **B** Kaplan–Meier overall survival curves of TCGA primary breast cancer dataset according to the cluster assignment. **C** Kaplan–Meier overall survival curves of TCGA primary Luminal breast cancer cohort determined by comparing Cluster 1 vs Cluster 2 vs Cluster 4. **D** Kaplan–Meier overall survival curves of TCGA primary Luminal breast cancer dataset determined by comparing Cluster 2 vs Cluster 4. **E** Gene ontology enrichment analysis of the differentially expressed genes between Cluster 4 and Cluster 2

The clustering analysis identified four groups that show a different magnitude of onco-signatures activation (Fig. 2A).

### The four identified breast cancer clusters show a different outcomes in terms of overall survival

Clustering analysis divided the TCGA primary breast cancer cohort into four groups (Fig. 2A), showing different outcomes in terms of overall survival (OS) (Fig. 2B). Cluster 4, enriched in Luminal A breast cancer subtypes (see Additional file 2: Fig. S1), showed a better OS with respect to the other groups (Fig. 2B). Conversely, Cluster 2 was characterized by the worse prognosis (Fig. 2B), although it was also mainly composed of Luminal tumors (Luminal A and Luminal B together; Additional file 2: Fig. S1). Cluster 1, enriched by Luminal subtypes, and Cluster 3, composed almost exclusively of Basal-like cancers, which are classically defined as the more aggressive subtypes of breast cancer, instead, showed medium survival with respect to Cluster 2 and the Cluster 4 (Fig. 2B). To verify whether the prognostic difference between clusters is specifically driven by the amount of Luminal tumors in the various groups, we decided to re-run the survival analysis only using the Luminal breast cancer cohort. As expected and according to the previous analysis depicted in Fig. 2A, B, the cancers belonging to the Cluster 4 remained associated with prolonged survival using only the BRCA-TCGA Luminal type dataset (Fig. 2C). The analysis also confirmed the worse survival for Luminal samples enriched in Cluster 2, while Cluster 1’s Luminal tumors showed an intermediate prognosis compared to Cluster 4 and Cluster 2 (Fig. 2C). Of note, due to the low number of Luminal cases in cluster 3 (N = 6), it was excluded both from this and the further analyses.

### A subset of luminal tumors shows a OXPHOS phenotype

**Fig. 3:**
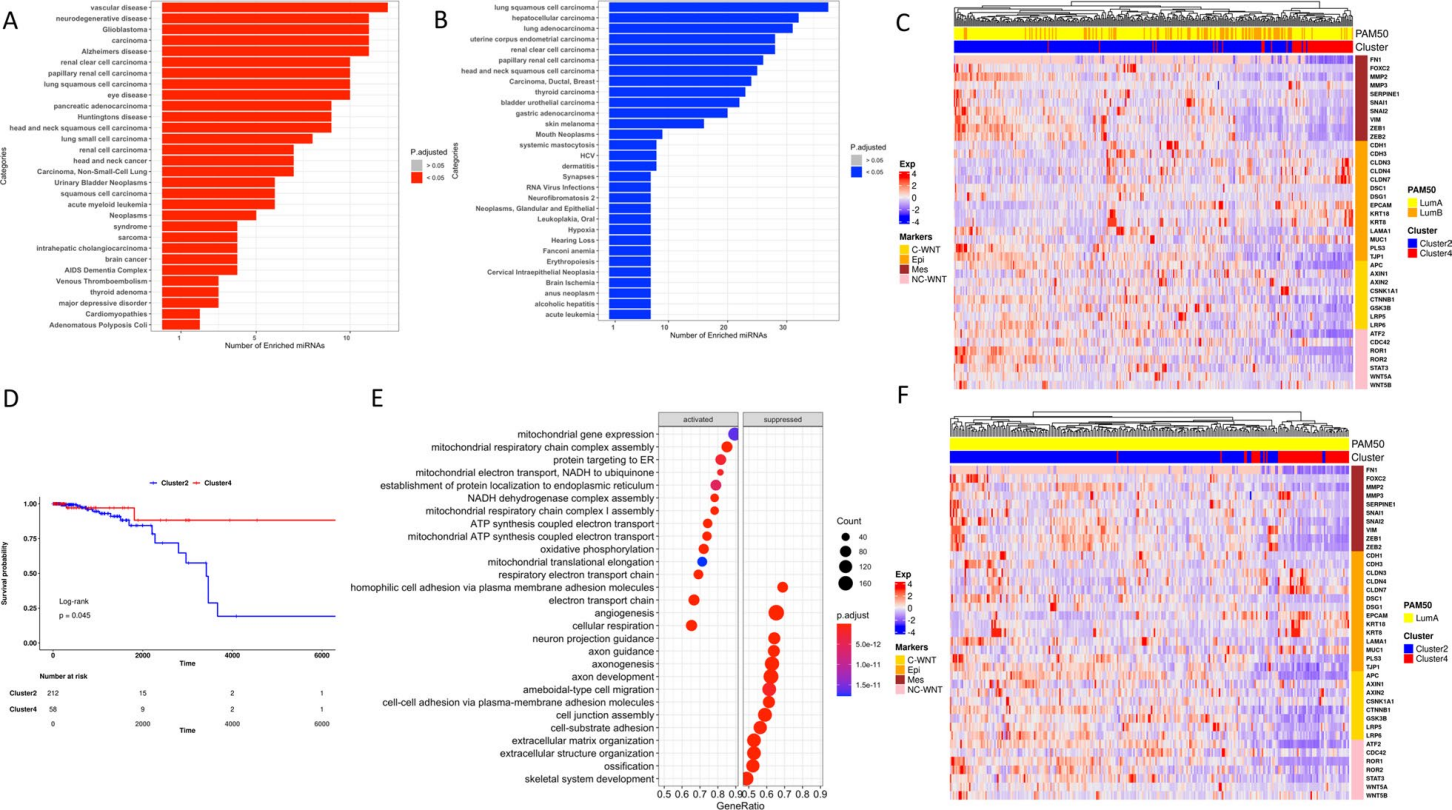
EMT markers together with Wnt/β-catenin signaling pathway members segregate Luminal breast tumors according to the cluster-assignment. **A**, **B** Results of the over-representation analysis performed by using the up-regulated (**A**) and the down-regulated (**B**) microRNAs derived by the differential expressed miRNA analysis. **C** Heatmap showing expression levels of the more representative epithelial–mesenchymal markers (Epi = epithelial, Mes = mesenchymal) and WNT (C-WNT = canonical WNT, NC-WNT = non canonical WNT) signaling pathway members in Cluster 2 and Cluster 4. The color key from blue to red indicates low to high gene expression, respectively. **D** Kaplan–Meier overall survival curves of TCGA primary Luminal A breast cancer cohort determined by comparing Cluster 2 vs Cluster 4. **E** Gene ontology enrichment analysis of the differentially expressed genes between Cluster 4 and Cluster 2 computed using only the Luminal A cohort. **F** Heatmap showing expression levels of the more representative epithelial–mesenchymal markers (Epi = epithelial, Mes = mesenchymal) and WNT (C-WNT = canonical WNT, NC-WNT = non canonical WNT) and signaling pathway members in Cluster 2 and Cluster 4 Luminal A samples

To evaluate the biological functions and the molecular features that could potentially explain the observed difference in survival outcomes across the Luminal breast cancer patients belonging to the identified clusters, we performed a comparative analysis between the samples that make-up to more extreme phenotypes, i.e. Cluster 4, characterized by the better survival, and Cluster 2, with poorer outcome (Fig. 2D). We performed a differential gene expression analysis between Cluster 4 versus Cluster 2 and, according to the cutoff value of |Log2FC| ≥ 1.5 and false discovery rate (FDR) < 0.05, we found 645 differentially expressed genes (DEGs; 243 up- and 402 down-regulated genes. DEGs’ list is provided as Additional file 3: Table S2). Gene ontology (GO) enrichment analysis showed that the biological process enrichment terms of up-regulated DEGs were mainly associated with mitochondrial respiration functions, electron transport chain, and oxidation–reduction processes (Fig. 2E). Conversely, the most significantly down-regulated genes were involved in GO terms related to brain-tissue functions, extracellular matrix organization, and cell adhesion (Fig. 2E). MiRNA differential expression analysis identified 60 deregulated miRNAs (16 up- and 44 down-regulated microRNAs; |Log2FC| ≥ 1 and FDR < 0.05). Over-representation analysis of the 16 up-regulated miRNAs captured the enrichment categories associated with cancer, including brain cancer, and neurodegenerative disorders (Fig. 3A). MiRNA set enrichment analysis of the 44 down-regulated microRNAs, instead, revealed enrichment substantially linked to cancer, and in particular with lung squamous cell carcinoma (adjusted p-value = 1.94e−15; Fig. 3B).

### The tumor-suppressive miR-135a-5p is up-regulated in mitochindiral luminal samples, and it could partially explain its favorable phenotype

Luminal breast cancers belonging to Cluster 4 showed a significant up-regulation in miR-135a-5p expression (log2FC = 2.9 and FDR = 3e−99) when compared with Cluster 2’s Luminal tumors (the list of DEmiRs is provided as Additional file 4: Table S3). Since it is well known that miR-135a-5p is a regulator of breast cancer epithelial–mesenchymal transition (EMT) acting by the Wnt/β-catenin signaling pathway [28, 29], we compared the transcriptional levels of both EMT and canonical and non-canonical WNT signaling pathway markers in Cluster 4 versus Cluster 2. As it is depicted in Fig. 3C, the EMT and the WNT signaling pathway members perfectly segregate the Luminal samples according to the cluster classification. Moreover, the two clusters show opposite profiles of expression of particular markers, with a heightened expression of epithelial members in Cluster 4 and elevated expression of mesenchymal markers and WNT members in Cluster 2 (Fig. 3C).

### “Mitochondrial” luminal A tumors are characterized by a more favorable outcome independently of their intrinsic molecular classification

As the percentage of Luminal B samples, known to be more aggressive than Luminal A tumors, is higher in Cluster 2 compared to Cluster 4 (respectively 29% and 12%), we re-run all analyses removing Luminal B participants in order to reduce the potential bias arising from molecular subtyping. Surprisingly, superimposable results were obtained by running the analysis only on the Lumina A cohort as depicted in Fig. 3D–F.

### The downregulation of miR-135a-5p in Cluster 2 could be, partially, explained in terms of TDMD

**Fig. 4:**
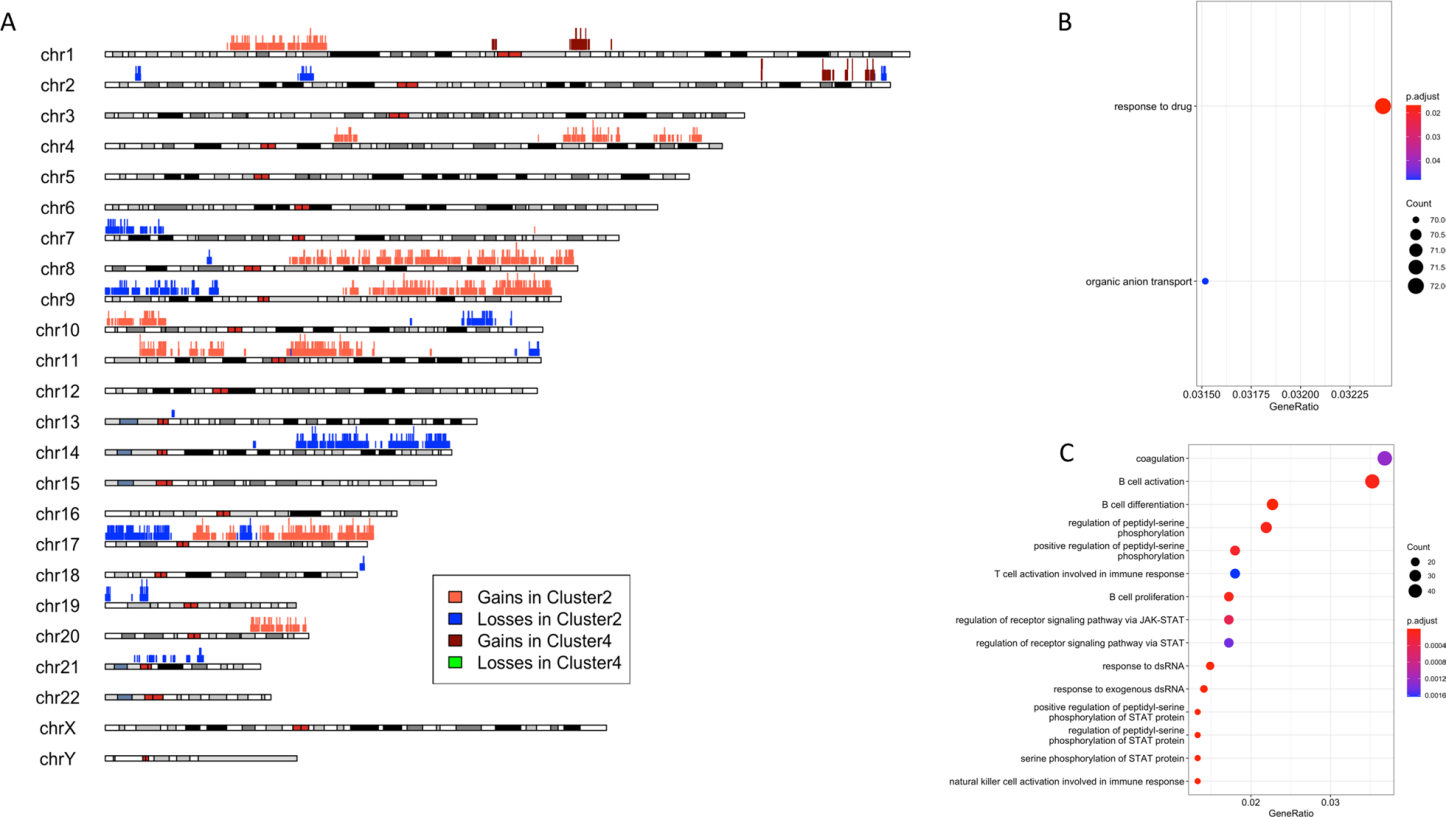
Copy number alterations characterizing Cluster 2 and Cluster 4 breast cancer groups. **A** Idiogram of autosomal genes significantly and differentially altered in Cluster 2 (deleted in blue and amplified in red) and in Cluster 4 (deleted in green and amplified in red-tomato). **B**, **C** Functional enrichment of genes significantly and differentially amplified (**B**) and deleted (**C**) in Cluster 2. No enrichment terms for the significant genes differentially altered in Cluster 4

**Fig. 5:**
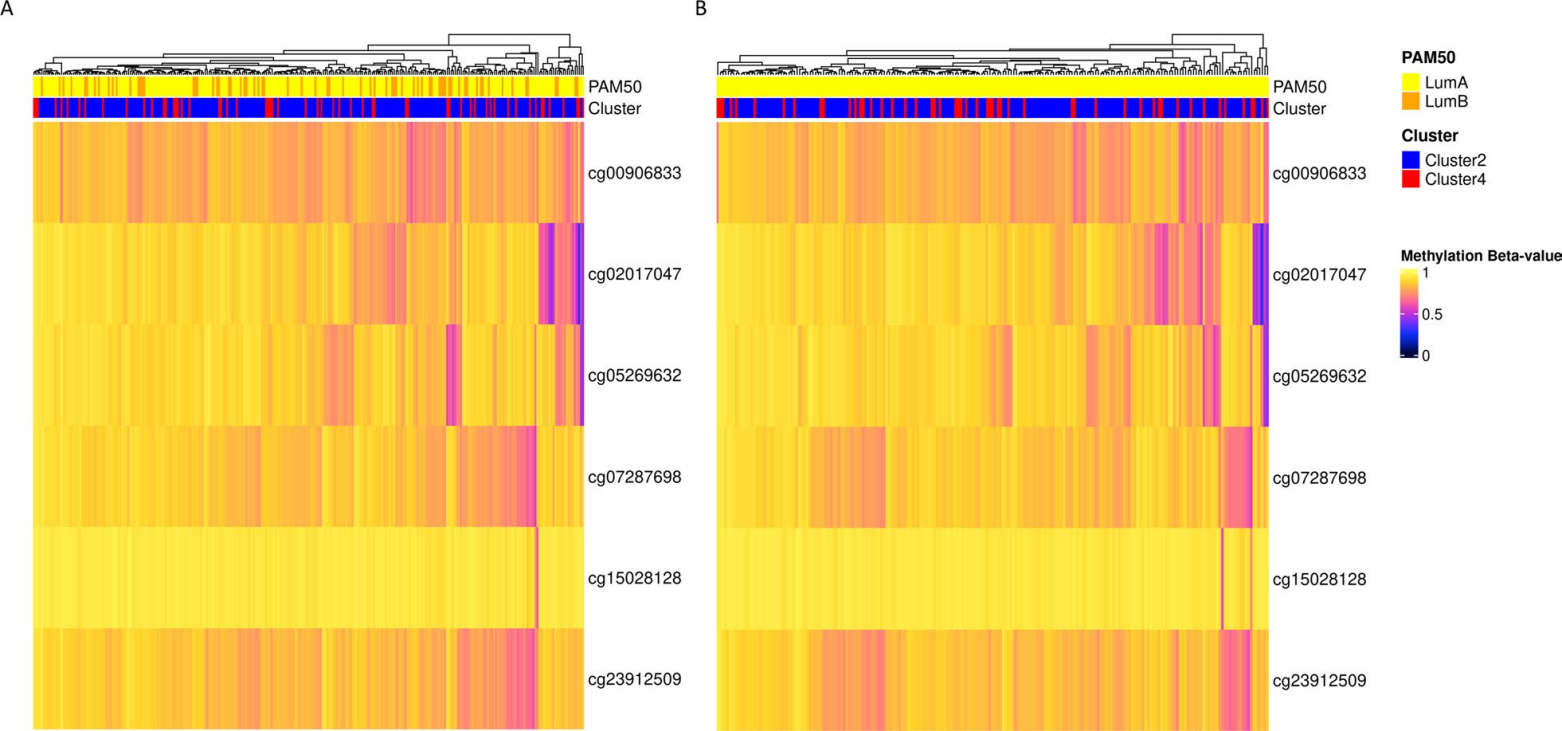
Analysis of promoter methylation of miR-135a genes in Cluster 2 and Cluster 4 Luminal samples. **A**, **B** Methylation status of the six site-specific probes associated to MIR135A genes in TCGA Luminal (**A**) and Luminal A (**B**) breast cancer cohorts. Rows represent array probes for MIR135A1 and MIR135A2 genes, columns represent samples. Cells are colorized according to level of methylation (black = hypomethylated, yellow = hypermethylated). The features of each sample, respectively the cluster assignment and the molecular subtype classification (PAM50), are annotated in the top bars

**Fig. 6:**
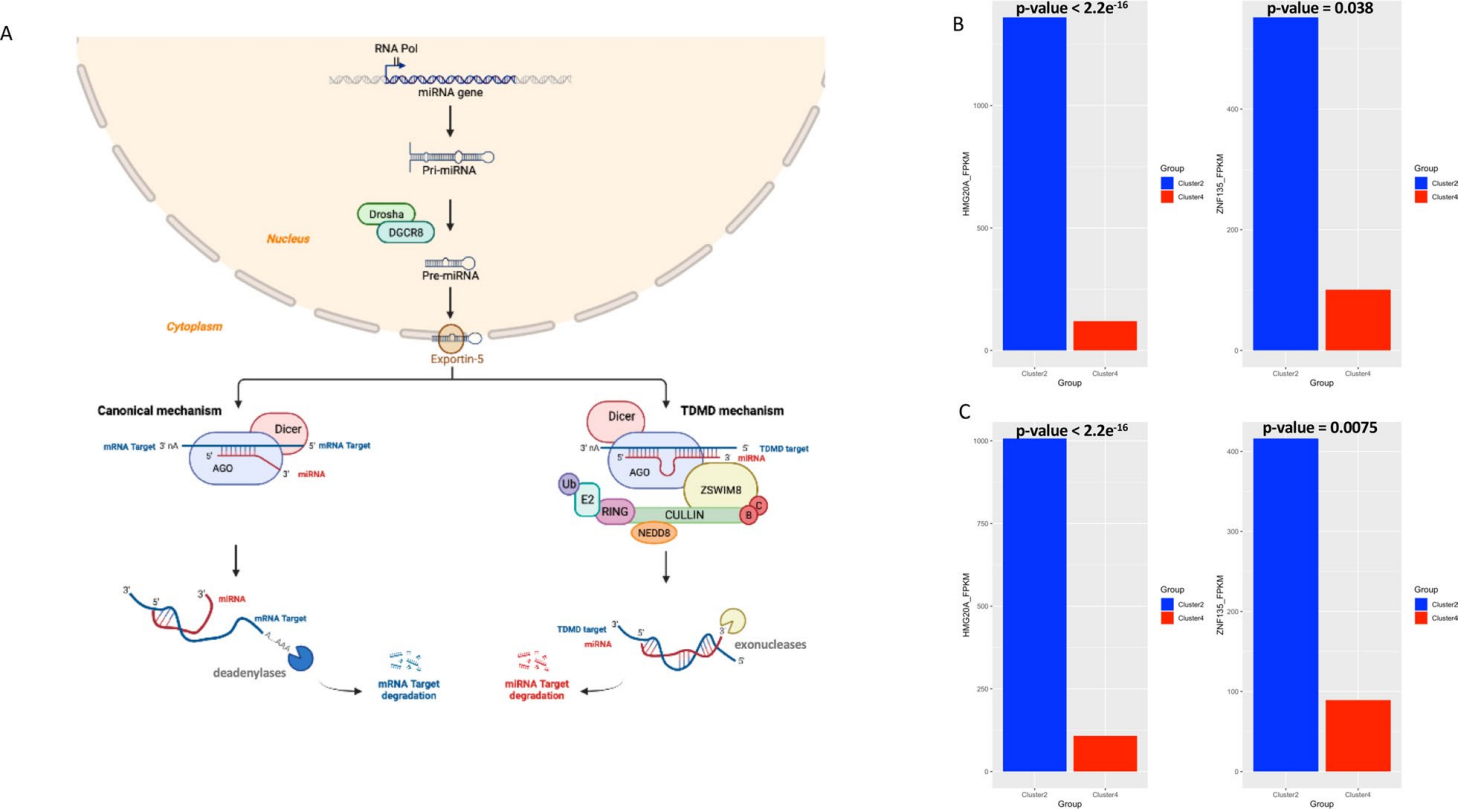
The TDMD hypothesis may explain the different expression level of hsa-miR-135a-5p between Cluster 2 and Cluster 4. **A** Schematic representation of the microRNA biogenesis (top panel) and of the two different types of miRNA:target interactions: canonical mechanism (bottom left panel) and TDMD (bottom right panel). MiRNA biogenesis starts in the nucleus, where the enzyme RNA Polymerase II (Pol II) transcripts the miRNA gene into a primary miRNA (pri-miRNA). Next the endonuclease Drosha together with its cofactor DGCR8 cleaves the pri-miRNA into smaller stem-looped structure known as precursor miRNA (pre-miRNA), which is exported to the cytoplasm by the Exportin-5. In the cytosol it is processed by Dicer, a RNAse endonuclease, to form a 21–24 nucleotides long miRNA duplex. The miRNA duplex is then loaded into an Argonaute (AGO) protein to form the miRNA-Induced Silencing Complex (miRISC). Only one strand, the miRNA guide strand, is retained in AGO, while the other strand, known as the passenger strand, is degraded. At this point the miRNA’s fate depends on the degree of complementarity between the miRNA and its target mRNA. A seed-dependent miRNA binding induces the degradation of the mRNA target (canonical mechanism). Conversely, a perfect pairing between the miRNA and its target mRNA characterized by a centered region of few mismatches can induce the degradation of miRNA via TDMD mechanism. **B**, **C** Expression levels of the two predicted high confident TDMD targets for hsa-miR-135a-5p (i.e. HMG20A and ZNF135) in Cluster 2 and Cluster 4, measured both in Luminal (**B**) and in Luminal A (**C**) cohorts. The transcript expression levels are significantly (Wilcoxon test, p < 0.05) higher in Cluster 2 compared to Cluster 4, according to a potential TDMD mechanism

In order to try to find molecular features related to such a different survival outcome between Luminal tumors of Cluster 2 and Cluster 4, additional analyses were performed. When we analyzed GISTIC processed SNPs data, we found that although the proportion of cases showing gains and/or losses was comparable across the two analyzed groups, Cluster 2 globally showed a more stable pattern of copy number alterations compared to Cluster 4 (see Additional file 5: Fig. S2). Instead, analyzing the frequencies of losses and gains between the two clusters at the single-gene level we found 3118 amplified and 1757 deleted genes significantly enriched in Cluster 2 versus Cluster 4, and just 130 amplified genes significantly enriched in Cluster 4 versus Cluster 2 (Fig. 4A). Interestingly, GO enrichment analysis of genes more frequently altered in Cluster 2 revealed that the 569 amplified genes were involved in functions and processes linked to response to drug and organic anion transport (Fig. 4B), whereas the 292 deleted genes showed enrichment in GO related to immune response and regulation of receptor signaling pathway via JAK-STAT (Fig. 4C). Conversely, we did not find enriched GO terms for the 23 frequently amplified genes identified in Cluster 4. Furthermore, the CNVs of the two genes coding for miR-135a-5p transcript, respectively MIR135A1 and MIR135A2 genes, were compared by chi-squared test between the two clusters, but neither gene showed significantly (p-value < 0.05) higher frequencies of alteration in Cluster 2 (MIR135A1: deleted and amplified, respectively, in the 32.1% and in the 6.4% of samples; MIR135A2: deleted and amplified, respectively, in the 12.2% and in the 21.0% of samples) or Cluster 4 (MIR135A1: deleted and amplified, respectively, in the 23.4% and in the 7.8% of samples; MIR135A2: deleted and amplified, respectively, in the 9.4% and in the 23.4% of samples). Similarly, on analysis of methylation beta values, we found that the promoter regions of MIR135A1 and MIR135A2 genes did not show different methylation status between the two clusters (Fig. 5A, B). Next, in order to understand if the difference of expression of miR-135a-5p between Cluster 2 and Cluster 4 could be explained at the genomic level in terms of transcriptional regulation, we tried to find candidate transcription factors (TFs) binding sites in DNA promoter sequences of MIR135A1 and MIR135A2 genes using an approach based on sequence matching. From this analysis, we found 122 potential transcription factors binding sites enriched in MIR135A promoter genes (for more details related to the list of putative transcription factor binding sites in both human MIR135A promoter elements see Additional file 6: Table S4). The intersection between the 122 candidate TFs and the DEGs list revealed an overlap of just 4 genes (ZNF354C, NFATC2, NFIC, and EHF), all significantly down-regulated in Cluster 4 versus Cluster 2. We next investigated whether the difference between the expression levels of miR-135a-5p in Cluster 4 and Cluster 2 could be induced by a post-transcriptional event. Since it is currently known that many RNA transcripts are able to trigger the degradation of microRNAs via the mechanism known as target-directed miRNA degradation (TDMD) [30–33], we tried to query TDMDfinder database (http://213.82.215.117:9999/TDMDfinder/index.php), the first and unique catalog of miRNA:TDMD-target predictions [34], to search potential TDMD target degraders of miR-135a-5p. An overview of the TDMD mechanism is depicted in Fig. 6A. TDMDfinder tool predicted two highly confident TDMD targets for miR-135a-5p, i.e., the Zinc Finger Protein 135 (ZNF135), involved in cytoskeleton organization and cell morphogenesis processes [35], and HMG20A (High Mobility Group 20A) gene, which is known both to play a role in neuronal differentiation and to be an essential factor for the development of the mesenchymal phenotype [36]. When we evaluated the expression levels of the two potential TDMD inducers in Cluster 2 and Cluster 4, we found that they were significantly higher in Cluster 2 compared to Cluster 4 (Wilcoxon test p-value < 0.05, Fig. 6B, C), according to a potential TDMD effect.

### The two different metabolic luminal breast cancer groups also show marked differences in terms of tumor microenvironment (TME) state

**Fig. 7:**
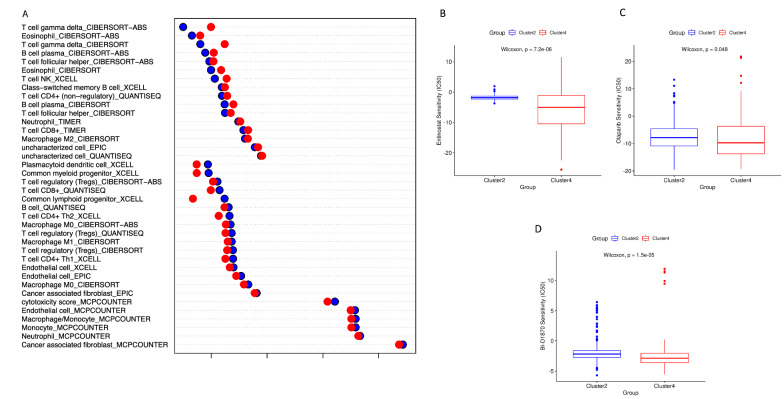
Exploration of the differences in immune infiltration and chemotherapy response. **A** Bubble chart illustrating the mean abundance of the more significant (Wilcoxon test, p < 0.01) tumor-infiltrating immune and cancer cell types between Cluster 2 (blue dots) and Cluster 4 (red dots). For each data point the mean values are represented in log10 form. **B**–**D** Evaluation of the sensitivity of chemotherapy drugs between the Cluster 2 Cluster 4 based on the IC50 values of Entinostat (**B**), Olaparib (**C**), and BI-D1870 (**D**), for Luminal breast cancer patients

The abundance of different tumor-infiltrating immune and cancer cell types was evaluated for the Luminal tumors of the two groups of interest, i.e., Cluster 2 and Cluster 4, by using the six immune deconvolution methods provided by TIMER2.0 webtool. As depicted in Fig. 7, the Luminal tumors enriched in Cluster 4 showed higher infiltration of immune effectors like γδ T cells, T follicular helper cells (Tfh), Macrophages M2, natural killer T (NKT) cells, Eosinophils, Neutrophils, plasma B cells, and non-regulatory CD4 T cells.

Conversely, the tumor microenvironment associated with Luminal cancers of Cluster 2 showed a more high infiltration of regulatory T cells (Tregs), Plasmacytoid Dendritic Cells (PDCs), Common Lymphoid Progenitors, Macrophages M0 and M1, CD4+ Th1 cells, and CD4+ Th2 cells, as well as, a higher cytotoxicity score. In addition, we also used pRRophetic algorithm to estimate the IC50 values of 138 anticancer drugs in the Luminal samples enriched in Cluster 2 and Cluster 4. Interestingly, Cluster 2’s Luminal tumors demonstrated much higher sensitivity to treatment with three anticancer drugs, i.e. Entinostat (or MS-S75), Olaparib (or AZD2281), and BI-D1870 (Wilcoxon test, p < 0.05; Fig. 7B–D), respect to Cluster 4 Luminal samples.

## Discussion

In this study, we presented a novel pipeline of analysis to identify robust onco-signatures potentially able to predict disease outcome in cancer patients. Using a pan-cancer discovery set of 9107 primary tumor samples together with respective matched mutational data, and a list of known cancer-related genes, we identified 105 onco-signatures, each one composed by a group of distinct marker genes. Aiming to investigate the predictive power of the 105 onco-signatures in breast cancer disease, the Cox proportional hazard regression model was constructed using the TCGA BRCA gene expression dataset, identifying 28 BRCA survival-associated onco-signatures. Next, by performing a gene-set enrichment analysis followed by an unsupervised hierarchical cluster analysis of NESs we identified four discrete breast cancer groups of clinical relevance. Our approach has successfully stratified the Basal-like breast tumors but not the Luminal tumors, who showed high diversity in terms of overall survival across the different clusters. Confirmation of the prognostic difference observed for Luminal cancers enriched in the four identified groups encouraged in-silico molecular analyses to discover the associated genetic variables, which showed profound differences between the more extreme Luminal phenotypes (i.e., Cluster 2 and Cluster 4) with respect to differential gene expression, CNV status, and activation of oncogenic signatures.

Differential gene expression analysis between the two groups of interest provided additional details on their molecular status. Cluster 4 Luminal tumors showed up-regulation of genes linked to mitochondrial respiration and oxidative phosphorylation. In contrast, Cluster 2 Luminal tumors displayed enrichment of genes involved in the development of central nervous system components, and extracellular matrix organization. Looking at genomic imbalances related to the two clusters we also noted that at the genomic level Cluster 4 tumors exhibited a higher frequency of amplifications and deletions as compared to Cluster 2 samples, although at genic-level these imbalances affected a higher number of genes in Cluster 2. Interestingly, we found that the Cluster 2 tumors were enriched in amplifications borne by genes involved in several metabolic processes. On the other hand, the deleted genes in Cluster 2 are involved in immune-cell suicide mechanisms. Although it is not possible to define the impact that such molecular events might have caused, some findings captured our attention. Currently, cancer is considered both a proliferative disorder and a metabolic disease [37, 38]. It is well known that different breast cancer subtypes have distinct bioenergetic and metabolic phenotypes which are associated with different survival outcomes [38]. For example, the Luminal-like tumors present a higher mitochondrial respiratory rate compared to the more metastatic Basal-like cancers that require, instead, an intensive glycolytic flux together with the reduction in OXPHOS processes [38]. In line with our findings, it is possible to hypothesize that an altered mitochondrial metabolism in Cluster 2 may be linked to an unfavorable survival outcome for the Luminal tumors enriched in this group, conferring them a growth advantage. In addition, since recent studies have demonstrated that mitochondrial metabolic processes are modulated by tumor cell-microenvironment [39–42], the specific deletion of genes involved in immune-cell death pathways that we found in Cluster 2 could reveal a potential cross-talk between mitochondrial dysfunction and the cancer immune microenvironment of this cluster.

Differential miRNA expression analysis provided additional details on the biology of the two subgroups of Luminal tumors in comparison, identifying the hsa-miR-135a-5p as the most up-regulated microRNA enriched in the cluster with the longer overall survival, i.e., Cluster 4. It has been demonstrated that mammary tumors display an altered expression of the microRNAs, many of whom function as oncogenes or tumor suppressors and modulate a variety of biological processes such as cell proliferation, migration, invasion, metastasis, apoptosis, differentiation, and cellular metabolism [43, 44]. Dysregulation of miR-135a-5p has been described in several cancer types [45–47]. Studies on the biological function of miR-135a in cancer have shown that it can play both oncogenic and antitumor roles depending on the cancer type, although it has been described that in breast cancer miR-135a-5p overexpression is able to inhibit EMT by acting through Wnt/β-catenin signaling pathway [28, 48]. In addition, other investigations have also linked mitochondrial activity to epithelial–mesenchymal transition in breast cancer, suggesting that the down-regulation of CDH1 and CTNNB1 in triple-negative breast tumors is correlated to a significant decrease in mitochondrial respiration [49]. Interestingly, our findings confirm the notion that miR-135a-5p is a potential tumor suppressor in breast cancer disease and could be used as a potential prognostic marker for this pathology.

Recent studies have described a special avenue for the downregulation of miRNAs, named target-directed microRNA degradation (TDMD), which induces the direct degradation of miRNAs [30–33, 50–52]. Although so far there are few studies on the TDMD mechanism, Simeone et al. [34] have performed the first computationally prediction of TDMD inducers in mammalian genomes making available their prediction in TDMDfinder webtool (http://213.82.215.117:9999/TDMDfinder/index.php). Investigating the possible cause that could explain the up-regulation of miR135a-5p in Cluster 4, or alternatively its down-regulation in Cluster 2, we queried TDMDfinder tool, which has predicted two high confident TDMD targets for hsa-miR-135a-5p. When we evaluated the expression levels of two potential TDMD inducers in our cohorts, we found that they are predominantly higher in Cluster 2 compared to Cluster 4. In addition, we also found that the biological functions of the two predicted TDMD-genes were associated to ontologies linked to neuronal tissues, where this mechanism was originally described as being particularly active, and pathways frequently altered in human tumors [30, 33]. Taken together these results suggest that the TDMD mechanism may be operative in Cluster 2.

To obtain better insights into the functional roles of the two different metabolic groups of Luminal breast tumors identified in this study, we also conducted analyses to evaluate both their immune infiltrating cell composition and the chemotherapeutic sensitivity to several drugs. Our findings showed that the tumor microenvironment of the Cluster 4 was characterized by a higher infiltration of anti-cancer effector cells, like the γδ T cells, T follicular helper cells, Macrophages M2, and natural killer cells, which are known to contribute to a good prognosis. Conversely, in the Luminal Cluster 2 samples, there was obvious immunosuppressive cells (e.g., the regulatory T cells) infiltration. This result may, at least partially, explain the favorable survival outcome observed in Cluster 4. In addition, in the present study we also showed that the Cluster 2's Luminal tumors were more sensitive to three chemotherapeutic compounds, i.e. Entinostat, Olaparib, and BI-D1870. Entinostat is an oral inhibitor of class I histone deacetylases (HDAC1) that shows a potent antiproliferative effect in breast cancer. Infact, mounting preclinical evidence suggests that it may have a role in immunogenic modulation inhibiting regulatory T cells and promoting tumor infiltration of lytic CD8+ T cells [53]. BI-D1870, instead, is a potent small molecule inhibitor of p90 ribosomal S6 kinases (RSKs) and it is widely used experimentally to revert the EMT phenotype in breast cancer cell lines since it can powerfully inhibit the growth of breast cancer cell lines [54, 55]. Olaparib is an oral poly(ADP ribose) polymerase (PARP) inhibitor that has promising antitumor activity in patients with aggressive forms of breast cancer disease. It, in fact, is the first treatment FDA (Food and Drug Administration)-approved specifically for BRCA mutation carriers with HER2-negative metastatic breast cancer [56–57]. Thus, the Luminal cancers characterized both by a low metabolic state and the Cluster 2-like genetic features group may also be valuable for clinical treatment, since our results demonstrated that this group of tumors was more sensitive to the different chemotherapeutic agents.

However, there are also limitations in our study. As in many studies on cancers, all omics data used in this work (CNV data, methylation data, mutational data, and miRNA expression data) could not be retrieved for additional datasets in order to perform a punctual in-silico validation. In addition, one of the more used breast cancer datasets has been created using microarray technology, making reproducibility of the onco-signatures very difficult. The lack of external independent validation results in a limit for our study. For this reason, future computational studies aiming to examine additional datasets, followed by biological validations useful to consolidate our findings are desirable.

## Conclusions

In conclusion, our study presents a valid and novel method based on gene set enrichment analysis to construct prognostic gene signatures in cancer. Analyzing in detail the TCGA breast cancer dataset, we demonstrated that our model is useful in predicting the prognosis of patients, and it is also able to stratify the cancer samples into more relevant subgroups. In particular, our onco-signatures were able to distinguish Luminal tumors characterized by different clinical and genetic features, as well as by a different metabolic state and a different tumor microenvironment. In addition, our analysis also provided potential therapeutic targets as well as candidate chemotherapeutic drugs for the improvement in treatment in Luminal patients with a lower mitochondrial activity which, of course, require further clinical confirmation.

## Supplementary Information

**Additional file 1: Table S1.** List of the 105 derived onco-signatures. List of 105 onco-signatures used in the paper.

**Additional file 2: Figure S1.** Circos plot clusters vs intrinsic molecular subtypes. Circos plot showing the distribution of different breast cancer subtypes (Basal = Basal-like, Her2 = Her2-enriched, LumA = Luminal-A, LumB = Luminal-B, Normal = Normal-like, Na = Not available) across the four identified clusters.

**Additional file 3: Table S2.** List of Differentially Expressed Genes (DEGs): Cluster 4 vs Cluster 2. List of genes exhibiting significant differential expression between Cluster 4 and Cluster 2.

**Additional file 4: Table S3.** List of Differentially Expressed miRNAs (DEmiRs): Cluster 4 vs Cluster 2. List of microRNAs exhibiting significant differential expression between Cluster 4 and Cluster 2.

**Additional file 5: Figure S2.** Cumulative CNV regions for Cluster 4 and Cluster 2. Visualization of CNV profiles in Cluster 4 (A) and Cluster 2 (B).

**Additional file 6: Table S4.** List of transcription factor binding sites in both human MIR135A promoter elements. List of putative transcription factors binding sites significantly enriched in MIR135A1 and MIR135A2 promoter regions.

## Data Availability

All the TCGA cancer data used in this study are available in GDC Data Portal (https://portal.gdc.cancer.gov/). All methods and materials used are described in the manuscript and they can be obtained from the corresponding authors upon reasonable request.

## Abbreviations

TCGA: The Cancer Genome Atlas
COSMIC: The Catalogue of Somatic Mutations in Cancer
ACC: Adrenocortical carcinoma
BLCA: Bladder urothelial carcinoma
BRCA: Breast invasive carcinoma
CESC: Cervical squamous cell carcinoma and endocervical adenocarcinoma
CHOL: Cholangiocarcinoma
COAD: Colon adenocarcinoma
DLBC: Lymphoid neoplasm diffuse large B-cell lymphoma
ESCA: Esophageal carcinoma
GBM: Glioblastoma multiforme
HNSC: Head and neck squamous cell carcinoma
KICH: Kidney chromophobe
KIRC: Kidney renal clear cell carcinoma
KIRP: Kidney renal papillary cell carcinoma
LGG: Brain lower grade glioma
LIHC: Liver hepatocellular carcinoma
LUAD: Lung adenocarcinoma
LUSC: Lung squamous cell carcinoma
MESO: Mesothelioma
OV: Ovarian serous cystadenocarcinoma
PAAD: Pancreatic adenocarcinoma
PCPG: Pheochromocytoma and paraganglioma
PRAD: Prostate adenocarcinoma
READ: Rectum adenocarcinoma
SARC: Sarcoma
SKCM: Skin cutaneous melanoma
STAD: Stomach adenocarcinoma
TCGT: Testicular germ cell tumors
THCA: Thyroid carcinoma
THYM: Thymoma
UCEC: Uterine corpus endometrial carcinoma
UCS: Uterine carcinosarcoma
UVM: Uveal melanoma
GDC: Genomic Data Commons
MiRNA: MicroRNA
DEG: Differentially expressed genes
MUT: Mutated
WT: Wild-type
NES: Normalized enrichment score
GO: Gene ontology
ORA: Over-representation analysis
DEmiR: Differentially expressed microRNAs
TSS: Transcription start site
IC50: Half-maximal inhibitory concentration
OS: Overall survival
FDR: False discovery rate
CNV: Copy number variation
TF: Transcription factor
TME: Tumor microenvironment
