# Supplementary figures and images for: Pan-cancer onco-signatures reveal a novel mitochondrial subtype of luminal breast cancer with specific regulators
Source: J Transl Med. 2023 Jan 30;21:55. doi: 10.1186/s12967-023-03907-z (PMC9885701; doi:10.1186/s12967-023-03907-z)

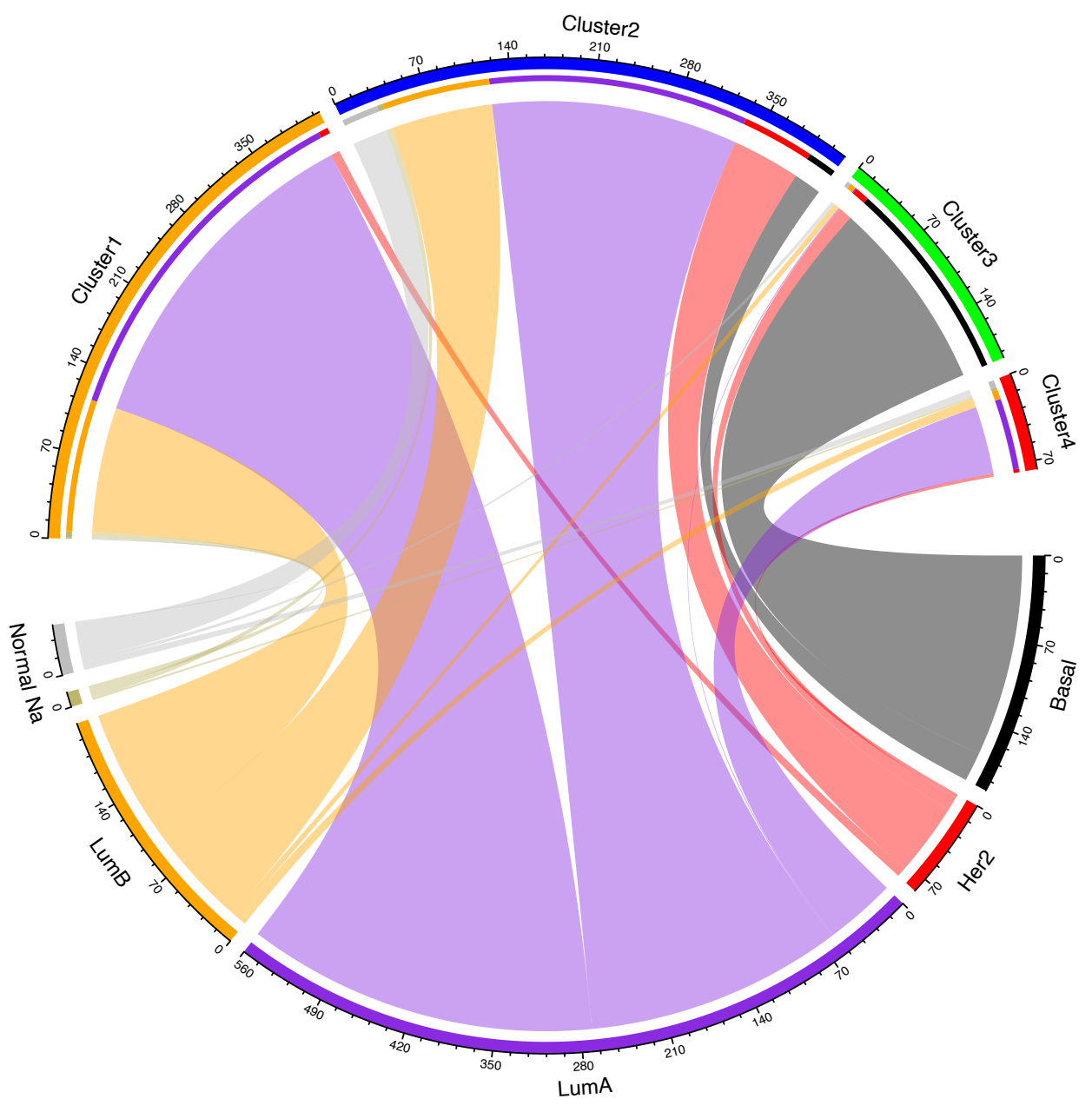

Supplement: Supplementary file 2 — Additional file 2: Figure S1. Circos plot clusters vs intrinsic molecular subtypes. Circos plot showing the distribution of different breast cancer subtypes (Basal = Basal-like, Her2 = Her2-enriched, LumA = Luminal-A, LumB = Luminal-B, Normal = Normal-like, Na = Not available) across the four identified clusters. [file 12967_2023_3907_MOESM2_ESM.pdf]

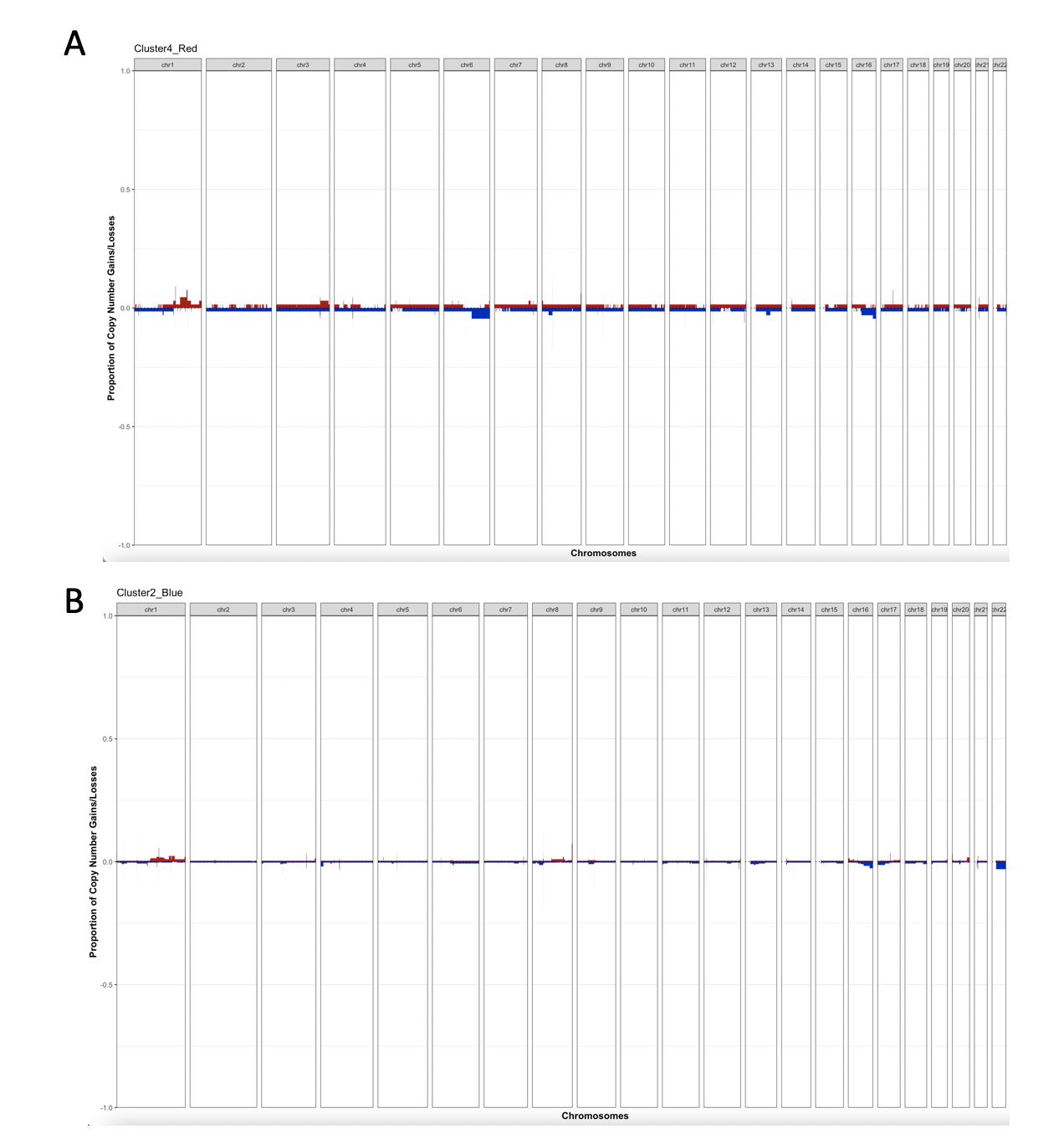

Supplement: Supplementary file 5 — Additional file 5: Figure S2. Cumulative CNV regions for Cluster 4 and Cluster 2. Visualization of CNV profiles in Cluster 4 (A) and Cluster 2 (B). [file 12967_2023_3907_MOESM5_ESM.png]
